# Annotations

**Published:** 1905-01-14

**Authors:** 


### ANNOTATIONS.

### The Question of Titles.

To judge from the correspondence columns of some of our contemporaries, there exists considerable discontent on the part of some members of the profession in regard to the general use of the term "doctor." Some who have no university degree claim the right to be distinguished equally with graduates, whilst certain of the latter protest against the adoption of the title by non-university men. Considered as an academic discussion, there is no difficulty in deciding that it is the possession of the M.D. degree which alone gives the strict right to the title, and that neither M.B.'s nor the licentiates of the medical and surgical corporations have any more claim to adopt the name of "doctor" than they have that of "marquis." But it is well known that it is common practice to describe any practitioner of medicine as a "doctor," and in many districts to address him by this title. Further, the rivalries of practice sometimes lead to a claim of superiority on the part of the graduate, and even to the direct depreciation of a fellow-practitioner's qualifications. It is out of these facts that there arise every now and then letters of protest and indignation which appear in some of the medical journals. Probably on careful investigation it would be found that the grumblers and the reformers are equally but a small class, and are either of somewhat junior experience or of inconsiderable professional activity. Most men of robust mind and healthy judgment discover after a few years' experience that the important thing in life in regard to success is the man himself and not the letters he can attach to his name, and the lay public are quite wide awake to this fact. Airs of superiority based upon the possession of a special hall-mark may be amusing in a boy, but in grown men they are simply ridiculous and suggest the absence of more substantial claims. The great body of the profession is too much concerned with serious duties and responsibilities to give much attention to names and titles. And the public is perfectly able to determine who is competent to render efficient aid in times of accident and sickness, and has little interest in the distinctions and phrases of professional classifications.

### Local Authorities and Health Problems.

The problems offered for solution by the facts of modern civilisation are both numerous and urgent, and in no aspect are they presented in a more acute form than in their relationship to the public health. It is, indeed, this relationship which forces many municipal questions into prominence and makes the conscience of the community sensitive to the appeals which they suggest. An extended publicity and an increased intolerance of unnecessary pain and suffering have bred a healthy impatience towards conditions condemned as antagonistic to the prosecution of vigorous bodily and mental development. A risk ever attached to this attitude of public opinion is that those in authority, feeling the pressure "to do something," adopt a policy which has the form of activity, but is destitute of the virtues of wisdom and discretion. Hence it is becoming increasingly important that local authorities shall be well provided with informed and competent advice. When the constitution of many of these bodies is remembered, and it is recalled how difficult and complex are many of the subjects presented to them for decision, some degree of anxiety is almost inevitable. Take, for example, one or two illustrations which have been attracting attention during the last few weeks. Widely spread over the country is the question of the unemployed; in one large city serious charges have been advanced against the honesty of the police force; in another the medical officer reports that the death-rate in one part of his district reaches the terrible ratio of 63 5 per 1,000, whilst the subject of labourers' cottages has secured a painful prominence under the policy of a certain rural council. That the settlement of such delicate questions as these is in the hands of men who in the majority of instances have private businesses of their own to manage is at least a matter for serious thought. Certainly it emphasises the proposition that the need for expert guidance is a very urgent one. The recognition of this necessity and the accurate determination of personal and official responsibility for municipal action may safely be presented as imperative conditions if the sanitary and other problems of organised communities are to be solved in accordance with the demands of justice and the public welfare.

### Medicine Measures.

Everyone is agreed that the existing practice of measuring medicine for administration to the patient in "spoonfuls" is inexact and unsatisfactory. But it is difficult to find a substitute as convenient and ready to hand as the domestic spoon, which therefore maintains itself in spite of repeated condemnation. The glass measures sold in shops for administering medicines are relatively expensive, offer opportunities for error, and, above all, they are brittle and easily come to grief. It is an experience of this latter quality which often moves domestic policy to return to the fashion of the older method. Such an objection will, it is to be anticipated, be fatal to Professor Cash's recent suggestion of a medicine-glass intended for the patient's use, and marked with only four graduations, which correspond respectively to 2·5, 5, 10, and 20 cubic centimetres. The idea is to facilitate prescribing in terms of the metric system by the provision of a measure to correspond to directions to the patient to take one, two, four, or eight "parts" of the mixture at each dose. Certainly the use of domestic measures must be considered by those who advocate the adoption of the metric system, but it may be questioned whether the suggestion of "part" as a domestic unit is quite a happy one. A label directing the patient to take so many "parts" of a mixture might easily be misconstrued and might lead to serious error. A satisfactory domestic or medicine-measure is a problem which awaits solution. Some years ago a series of small earthenware cups representing a teaspoon, dessertspoon, and tablespoon respectively were introduced, and personally we have found these very satisfactory. They were practically unbreakable and could be purchased for a few pence. But for some reason or other they do not seem to have obtained popularity. To be delivered from the risks and uncertainties of the household spoon would be a welcome experience. But we fear that the latest proposal has no chance of achieving this end.

